# Intramuscular injury between muscularis propria circular and longitudinal layers: a novel subtype of Sydney III deep mural injury?

**DOI:** 10.1007/s10151-023-02815-0

**Published:** 2023-06-13

**Authors:** V. Zimmer

**Affiliations:** 1 Department of Medicine, Marienhausklinik St. Josef Kohlhof, Neunkirchen, Germany; 2 Department of Medicine II, Saarland University Medical Center, Saarland University, Homburg, Germany

**Fig. 1:**
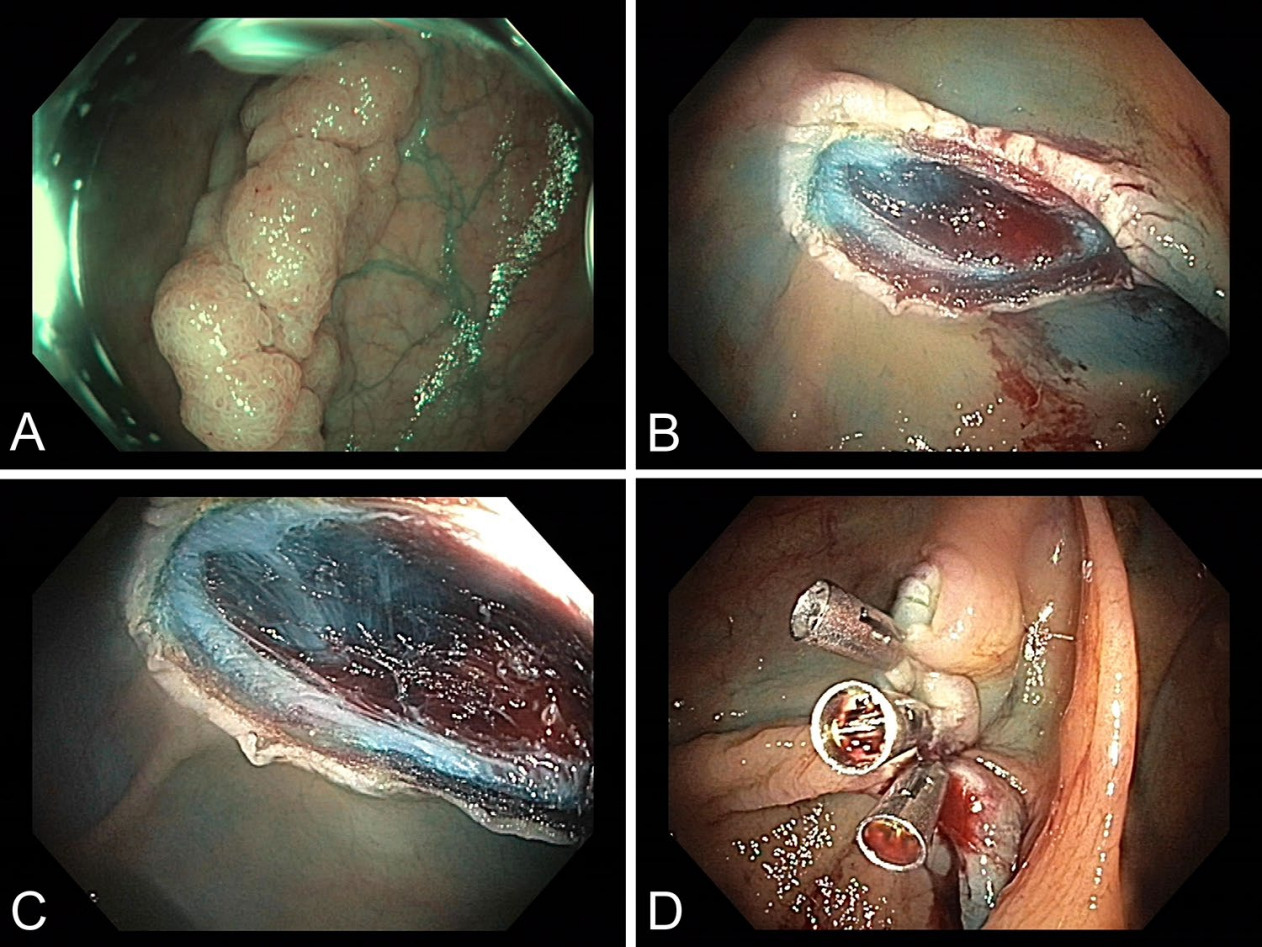
**a** Outpatient ileocolonoscopy revealed an estimated 22-mm polypoid lesion (granular-type laterally spreading lesion (LSL-G), Paris IIa, NBI International Colorectal Endoscopic (NICE) classification 2). **b** Visualization of the defect after en bloc mucosectomy indicated, apart from a peripheral vessel, a target sign involving the muscularis propria, yet without clear-cut perforation, compatible with a Sydney III deep mural injury (DMI). **c** A more detailed characterization demonstrated changes in the orientation of more superficial versus deeper muscle fibers, suggesting intra-mucscularis propria resection between the (inner) circular and (outer) longitudinal layers. **d** The defect was closed immediately with three clips resulting in complete closure and an uncomplicated post-interventional clinical course

Assessment of the defect after endoscopic resection of colorectal neoplastic lesions is standard in routine endoscopy and instrumental in identifying deep muscular injury (DMI) up to frank perforation in need of immediate endoscopic closure. DMI involving the muscularis propria, classified as a Sydney III lesion, usually implies complete muscularis propria transection. By contrast, intramuscular resection with potential implications in terms of complication risks, such as rate of post-electrocautery syndrome, has not been reported before [1] (Fig. 1).
